# Pericardial effusion as a manifestation of feline infectious peritonitis

**DOI:** 10.2478/jvetres-2026-0039

**Published:** 2026-08-03

**Authors:** Łukasz Mazurek, Stanisław Winiarczyk, Łukasz Adaszek, Katarzyna Michalak, Dorota Pietras-Ożga

**Affiliations:** 1 Department of Epizootiology and Infectious Diseases Clinic, Oncology Laboratory, Department of Epizootiology and Infectious Diseases Clinic, University of Life Sciences in Lublin, 20-950 Lublin, Poland; 2 National Veterinary Research Institute, 24-100 Puławy, Poland

**Keywords:** feline infectious peritonitis, pericardial effusion, cardiac tamponade, NT-proBNP, real-time PCR

## Abstract

**Introduction:**

Feline infectious peritonitis (FIP) is a fatal disease caused by feline coronavirus, most commonly associated with effusions in body cavities, while pericardial involvement remains rarely described.

**Material and Methods:**

A three-year clinical study (2021–2024) included 256 cats with PCR-confirmed effusive FIP, of which 37 presented fluid accumulation exclusively in the pericardial sac. Diagnosis was based on real-time PCR analysis of pericardial fluid, echocardiographic examination and measurement of serum N-terminal pro-B-type natriuretic peptide (NT-proBNP) concentrations.

**Results:**

Mutant feline coronavirus RNA was detected in pericardial fluid samples from all cats, with threshold cycle values ranging from 22 to 26 and highly specific PCR amplification confirmed by consistent melting temperatures (81.0–81.2°C). Ultrasonography revealed pericardial effusion in all 37 cats, with cardiac tamponade observed in 27% of cases and significantly higher NT-proBNP concentrations in affected individuals. Statistical analyses showed no significant associations between breed, age or sex and the occurrence of pericardial effusion, cardiac tamponade or NT-proBNP serum concentrations.

**Conclusion:**

Pericardial effusion and cardiac tamponade may represent under-recognised manifestations of effusive FIP. Routine echocardiographic evaluation and molecular analysis of pericardial fluid should be considered in cats with suspected FIP and cardiopulmonary signs.

## Introduction

Feline infectious peritonitis (FIP) is a viral disease caused by feline coronavirus (FCoV), a member of the order *Nidovirales*, family *Coronaviridae* and subfamily *Coronavirinae* (11, 18). Based on genetic structural analysis, this subfamily is divided into four genera, of which *Alphacoronavirus* is the genus of feline coronavirus (FCoV). The genome of FCoV has a length of 27–32 thousand base pairs (kbp), making it one of the largest RNA viruses (6). Approximately two-thirds of the FCoV genome encodes the replicase complex, *i.e*. enzymes responsible for the synthesis of viral RNA and for the replication and transcription of viral genetic material in an infected cell (4). Two FCoV biotypes are distinguished, which differ in virulence: the non-pathogenic intestinal biotype and the pathogenic FIPogenic biotype, which is a mutation of the intestinal strain (8). FIPogenic strains of feline coronavirus multiply intensively in the cells of the mononuclear and macrophage system, mainly in monocytes and macrophages. During the viraemia phase, they are distributed along with infected cells through the bloodstream to various organs and tissues (5). Monocytes, when migrating from small venous vessels to the extravascular space, release both virus particles and numerous pro-inflammatory cytokines, which initiates a local inflammatory response. This process results in the formation of granulomatous inflammatory lesions in internal organs, such as the liver, kidneys, lymph nodes, central nervous system and eyeballs. Such a course of the disease is referred to as the non-effusive (dry) form of FIP. If, however, granulomatous inflammation is accompanied by damage to the vascular endothelium and increased vascular permeability, a protein-rich fluid leaks into body cavities, most commonly into the abdominal and/or pleural cavities, but also into the pericardial sac (7). In an ultrasound image, this effusive (wet) form of FIP shows fluid in body cavities, which contains blood morphologic elements and conglomerates with fibrin characteristics (9). Recent years have seen a clear increase in the number of patients with wet FIP and characteristic fluid accumulation in the pericardial sac (17). The accumulation of fluid within this structure results in disturbances in cardiac muscle relaxation, increased pressure within the pericardial cavity (epicardium), and, in many cases, cardiac tamponade, *i.e*. a life-threatening condition requiring immediate medical and veterinary intervention (17). The study aimed to present the results of a three-year clinical study on the incidence of the wet form of infectious peritonitis in cats.

## Material and Methods

### Animals used in the study

The study involved 256 cats with the PCR-confirmed effusive form of FIP. In 176 cats, fluid accumulated exclusively in the abdominal cavity; in 43 cats in the chest cavity; and in 37 cats, it was only in the pericardial sac. These 37, in which echocardiography showed the presence of an increased amount of fluid (<17%), were qualified for further examination. The group comprised 17 females and 20 males aged between 5 months and 8 years (Table 1). The individuals under study were domestic animals receiving regular preventive treatment against ecto- and endoparasites. All the cats were fed commercial foods and had constant access to water. The disease was diagnosed based on the PCR results from pericardial fluid. During the study period of 2021–2024, all animals underwent echocardiographic examination, and blood samples were collected to determine feline N-terminal pro-B-type natriuretic peptide (NT-proBNP) concentration. None of the cats was treated prior to sample collection, because treatment could have influenced NT-proBNP concentrations. Potential differential diagnoses of pericardial effusion in cats, including neoplastic disease, bacterial or viral pericarditis, traumatic causes and idiopathic pericardial effusion, were carefully considered and excluded based on the absence of relevant clinical signs, characteristic echocardiographic findings, laboratory results and the overall clinical course of the disease.

**Table 1.** Characteristics of the studied cats with effusive feline infectious peritonitis and pericardial effusion

Item	Breed	Age	Sex	Presence of pericardial effusion	Presence of tamponade	Feline NT-proBNP concentration (pmol/L)
1	European mixed	3 years	Male		+	733
2	European mixed	4 years	Female	+		155
3	European mixed	11 months	Female	+		180
4	Maine coon	2 years	Male		+	230
5	British shorthair	8 months	Male	+		182
6	European mixed	5 years	Male	+		138
7	European mixed	7 years	Female	+		144
8	Sphynx	2 years	Female		+	380
9	European mixed	3 years	Female	+		99
10	European mixed	9 months	Male	+		80
11	Maine coon	1 year	Female	+		121
12	European mixed	2 years	Male	+		132
13	European mixed	5 months	Female		+	499
14	European mixed	7 years	Male	+		76
15	Siamese	1 year	Female	+		103
16	European mixed	5 years	Male	+		134
17	European mixed	5 years	Male		+	341
18	British shorthair	1 year	Female	+		133
19	European mixed	3 years	Female	+		240
20	Norwegian forest	2 years	Male	+		145
21	Sphynx	11 months	Female		+	589
22	European mixed	8 years	Male	+		74
23	Devon rex	9 months	Female	+		188
24	Maine coon	2 years	Male		+	204
25	European mixed	4 years	Female	+		311
26	Devon rex	3 years	Female		+	371
27	European mixed	1 year	Male	+		220
28	Devon rex	8 months	Female	+		169
29	European mixed	2 years	Female		+	435
30	European mixed	7 years	Male	+		80
31	European mixed	6 years	Female	+		180
32	Ragdoll	2 years	Female	+		209
33	European mixed	11 months	Male		+	530
34	European mixed	5 years	Female	+		90
35	European mixed	6 years	Male	+		330
36	Devon rex	2 years	Male	+		154
37	British shorthair	1 year	Female		+	484

1 NT-ProBNP – N-terminal pro-B-type natriuretic peptide

### Real-time PCR

A fragment of the *S* (spike) gene was amplified from pericardial fluid using a real-time PCR. Quantitative analysis of the studied gene expression was performed using 2 μL of a matrix containing 200 ng of cDNA derived from RNA (1). The real-time PCR was performed in 20 μL thin-walled tubes using the DyNAmo HS SYBR Green qPCR Kit (Finnzymes, Espoo, Finland), enabling highly specific qualitative and quantitative reactions. The PCR was performed using specific primers (sense 5'-CAATATTACAATGGCATAATGG-3' and antisense 5'-CCCTCGAGTCCCGCAGAAACCATACCTA-3') for the first reaction and second reaction (sense 5'-GGCATAATGGTTTTACCTGGTG-3', antisense 5’-TAATTAAGCCTCGCCTGCACTT-3'). The PCR cycling conditions were 30 cycles at 94°C for 60 s, at 50°C for 30 s and at 72 °C for 1 min, plus a 7-min extension at 72°C at the end of the reaction. Primer pairs were expected to generate a 598-bp product covering nucleotides 23,442–24,040 for the first PCR run and a 142-bp product covering nucleotides 23,451–23,593 (which includes deviant position 23,531 – mutation M1058L) for the second PCR run (3). The 20 μL volume of the initial reaction mixture contained 2 μL of DNA matrix, 7.2 μL of water, 0.4 μL of each primer (final concentration 50 pM), 10 μL of Master Mix containing the hot start version of the modified Tbr polymerase (*Thermus brockianus*), buffer for Tbr polymerase, dNTP, MgCl_2_ and SYBR Green 1 intercalating dye. Reactions were performed using a Rotor-Gene 3000 thermal cycler (Corbett Research, Sydney, NSW, Australia). The Ct value of the real-time PCR products generated on the complementary DNA matrix was determined for each reaction (the number of amplification cycles after which the fluorescence intensity of the resulting product exceeded the background fluorescence). To confirm the amplification specificity, the melting point of the PCR products was determined by gradually increasing the temperature and continuously measuring the fluorescence to generate a melting curve.

### Echocardiographic examination

Echocardiographic examinations were performed using an Esaote Mylab Class C ultrasound machine (Genoa, Italy) with a PA023 4–12 MHz phased array multi-frequency cardiac transducer on animals in a lying position. Each time, the structure and function of the cardiac muscle were assessed. Twodimensional imaging, M-mode (motion-mode) measurements and the pulsed/colour-coded Doppler technique were applied. Two-dimensional imaging included long-axis and short-axis projections from the right and left parasternal acoustic windows. Measurements of blood flow through the pulmonary artery and the aorta were performed. The diameters of the left atrium and the aorta were measured to determine the ratio of the aorta to the left atrium. The echocardiographic examination revealed free fluid in the pericardial sac, along with diastolic collapse of the right atrium and right ventricle, indicating cardiac tamponade (Fig. 1).

**Fig. 1.**
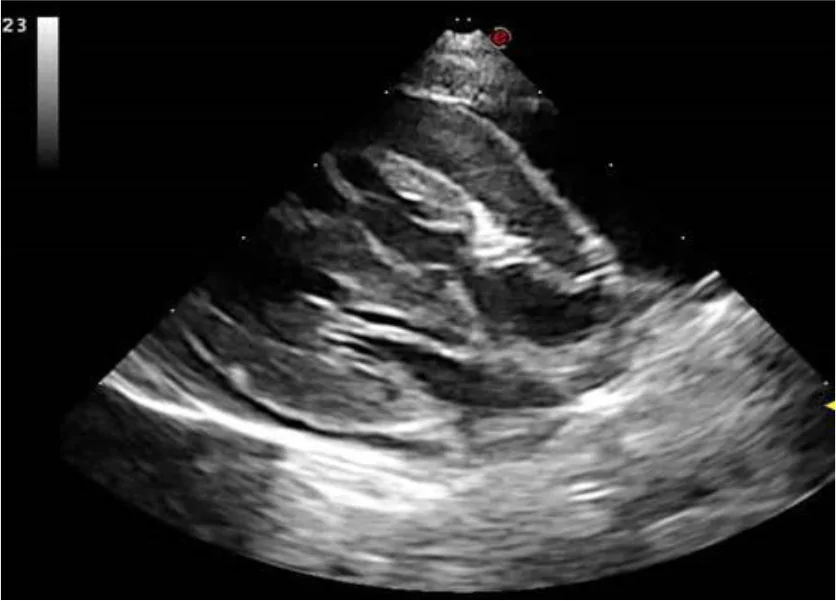
Echocardiographic image demonstrating feline cardiac tamponade with compression of the cardiac chambers due to pericardial effusion

### Measurement of the feline NT-proBNP concentration

This method was performed using a Vcheck analyser (Vetexpert, Warsaw, Poland) according to the manufacturer’s instructions. The physiological concentration of feline NT-proBNP was assumed to be within the range of <100 pmol/L. Considering the pressure on the cardiac muscle caused by the accumulated fluid, it was concluded that proBNP may be elevated because of cardiac muscle overload and dysfunction, which may be a complication of the effusive form of FIP.

### Statistical analysis

To assess the correlation between sex (male *vs* female), age (≤2 years *vs* >2 years), breed (purebred *vs* mixed-breed) and the presence of pericardial effusion (Fig. 2) or cardiac tamponade in cats, a chi-squared test of independence was applied to a 2 × 2 contingency table. Both variables were categorical, which justified the use of this method. Given the relatively small sample size (n = 37) and the occurrence of expected frequencies close to 5, Yates’ correction was used with the chi-squared test. This correction reduces the risk of overestimating statistical significance in small samples and in 2 × 2 tables, providing a more conservative assessment of the association between the variables under study. Statistical parameters are shown in Tables 2 and 3.

**Fig. 2.**
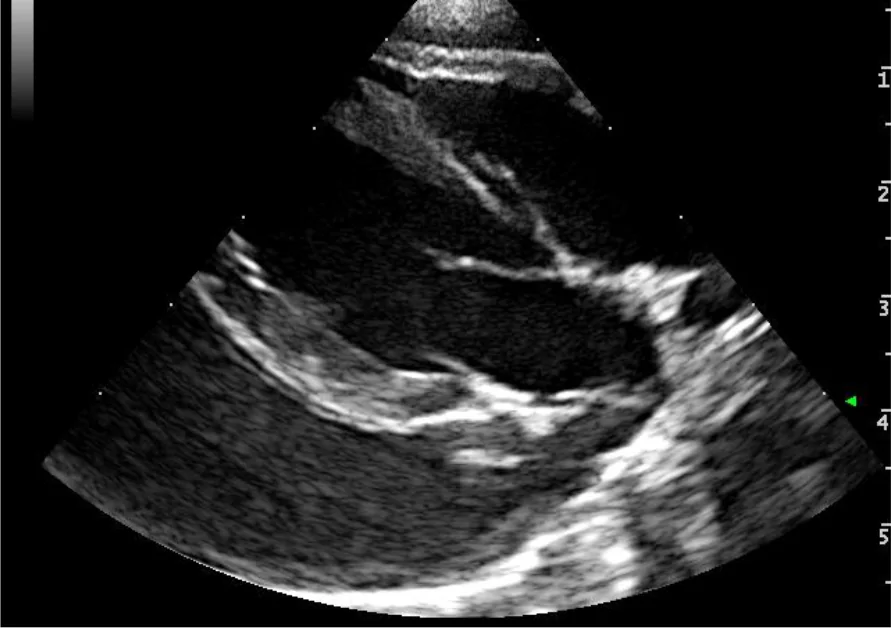
Echocardiographic image demonstrating pericardial effusion surrounding the heart of a feline patient

**Table 2.** Chi-squared test results for association between selected variables and the presence of pericardial effusion in cats

	Yates’ chi-squared	Yates’ P-value
Male *vs* female	0.104	0.747
Purebred *vs* mixed-breed	0.581	0.446
≤2 years *vs* >2 years	0.833	0.361

**Table 3.** Chi-squared test results for association between selected variables and the presence of cardiac tamponade in cats

	Yates’ chi-squared	Yates’ P-value
Male *vs* female	0.005	0.943
Purebred *vs* mixed-breed	0.581	0.446
≤2 years *vs* >2 years	0.833	0.361

Additionally, the possibility of differences in feline NT-proBNP levels (pmol/L) by sex, age, and breed was investigated. For this purpose, the normality of distributions within the study groups was initially assessed using the Shapiro–Wilk test (P-value < 0.05), and the results are shown in Table 3. Since the values in both groups did not follow a normal distribution (Shapiro–Wilk test: P-value < 0.05), the non-parametric Mann–Whitney U test (P-value < 0.05) was applied for comparison (Table 4).

**Table 4.** Basic statistical data and normality parameters

	Median concentration of feline NT-proBNP (pmol/L)	Mean concentration of feline NT-proBNP (pmol/L)	Shapiro-Wilk P-value
Male	154	223	0.0009
Female	184	254	0.0130
≤2 years	188	256	0.0018
>2 years	150	222	0.0016
Purebred	168	244	0.0065
Mixed-breed	188	236	0.0019

1 NT-proBNP – N-terminal pro-B-type natriuretic peptide

## Results

The presence of the mutant feline coronavirus genetic material was demonstrated in the fluid collected from all cats in the study group. A Ct value range of 22–26 was observed for individual samples. By assessing the melting point of the obtained amplicons, it can be concluded that the reactions had high specificity, which was confirmed by the similar melting point values of the PCR products, *i.e*. 81.0–81.2°C (in the case of enteric coronavirus, this value was lower, at about 83.3–83.5°C) (1).

The echocardiographic examination revealed the presence of pericardial effusion in all 37 cats under observation. In 10 cats (27%), fluid accumulation increased pericardial pressure and collapsed the right atrium, leading to cardiac tamponade. The animals showed signs of dyspnoea, exacerbated both at rest and during exercise, and of exercise intolerance, manifested by rapid fatigue, reluctance to play and reduced physical activity. The owners also reported rapid, shallow breathing, increased frequency of rest after short-term exertion, and, in some patients, paroxysmal coughing and periodic cyanosis of the mucous membranes.

The NT-proBNP serum concentrations in the patients under study ranged from 74 to 733 pmol/L (mean 238 pmol/L). Cats with tamponade (n = 10) exhibited markedly higher NT-proBNP concentrations (mean 437 pmol/L) compared to cats with pericardial effusion (mean 187 pmol/L). The group with the highest NT-proBNP concentration (>400 pmol/L) included individuals of various breeds: European mixed British shorthair, Sphynx and Devon rex.

Among 256 cats with effusive FIP confirmed by molecular testing, the presence of fluid in the pericardial sac was noted in 37 individuals. Among those 37 patients, in which fluid only accumulated in the pericardial sac, the European mixed cats were predominant (n = 23), followed by Maine coon (n = 3), Devon rex (n = 3), British shorthair (n = 3), Sphynx (n = 2), Siamese (n = 1), Ragdoll (n = 1) and the Norwegian forest cat (n = 1). The discussed form of the disease was most frequently observed in individuals aged 1–3 years (40.5%). Yates's chi-squared test showed no statistically significant differences (P > 0.05 in all comparisons). Therefore, no association was found between breed, age or sex and the occurrence of pericardial effusion or cardiac tamponade. Based on the Shapiro–Wilk test results (P-value < 0.05), nonparametric analysis using the Mann–Whitney U test was performed. No statistically significant differences in feline NT-proBNP concentrations were found between the groups with respect to breed, age or sex (P-value > 0.05) (Tables 4 and 5).

**Table 5.** Mann–Whitney U test parameters

	U	Mann–Whitney U P-value
Male *vs* female	138.0	0.337
Purebred *vs* mixed-breed	138.0	0.412
≤2 years *vs* >2 years	207.0	0.238

Based on the above groups, box and violin plots were generated (Fig. 3). The box plot presents the median, interquartile range and outliers in both groups, allowing assessment of data spread and variability. The violin plot additionally illustrates the distribution density of the results, highlighting areas with greater data concentration. In both cases, a partial overlap of values is observed, with no statistically significant differences between the group.

**Fig. 3.**
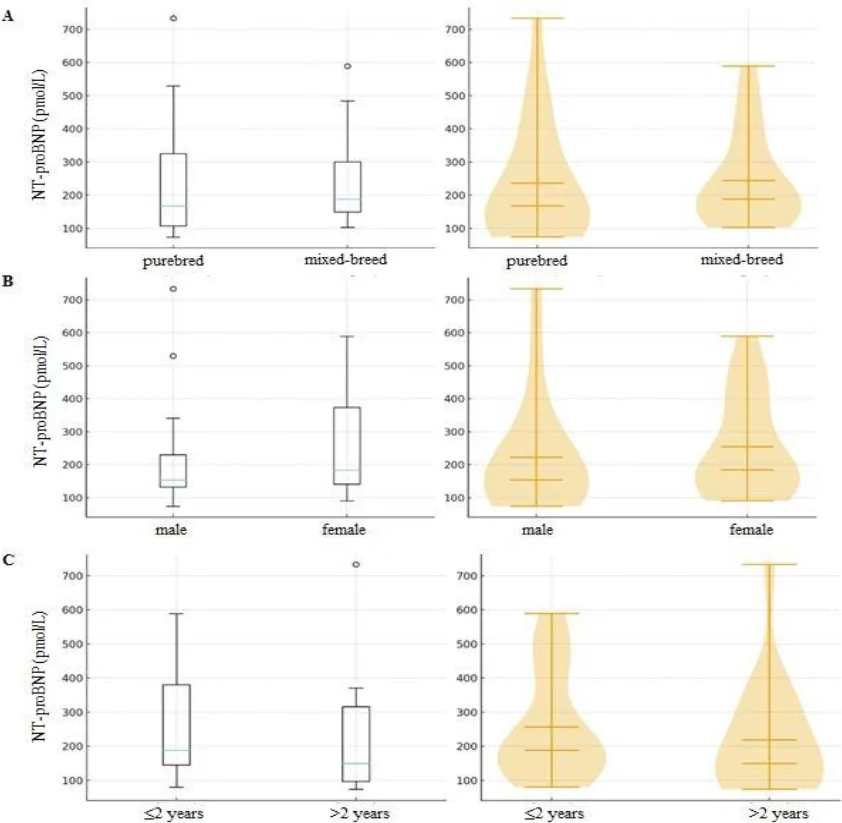
Box and violin plots showing the median, interquartile range and outliers for N-terminal pro-B-type natriuretic peptide (NT-proBNP) concentrations (pmol/L) in the studied groups of cats for: A) purebred *vs* mixed-breed**;** B) male *vs* female; C) ≤2 years *vs* >2 years

## Discussion

This study found no correlation between sex and disease occurrence. The literature indicates that animal age predisposes them to FIP, with the disease mostly affecting young individuals. This is related to the immune system not being fully developed in this age group. It is worth noting, however, that an increasing number of cases of the disease have recently been reported in cats over five years of age (13). The authors’ observations did not confirm any statistically significant differences in the development of FIP during which pericardial effusion developed between cats in the young groups and those in the adult groups. This discrepancy may result from the small group of animals under study, as well as the very nature of this symptom, which is not predominant in the effusive form of infectious peritonitis and may develop in older animals. These hypotheses, however, require further research. In the present study, the effusive form of FIP in which fluid accumulated exclusively in the pericardial sac was more frequently observed in European mixed cats. However, in this case, it must be considered that most of the animals under observation were European mixed cats, so this result may not be entirely reliable. Since European mixed cats form the majority of companion cats in Poland, it is natural that they will form the group in which FIP cases are most frequently reported, including only those for which fluid accumulates in the pericardial sac (13).

The mechanism of fluid formation in the pericardial sac in cats with FIP results from a complex inflammatory response initiated by the FIPogenic strain of FCoV, which multiplies in the monocytes and macrophages. Infected cells migrate to various organs, including structures surrounding the heart, to release pro-inflammatory cytokines (such as TNF-α and IL-1ß) and vascular endothelial growth factor. These mediators lead to increased vascular permeability and immune-mediated inflammation (type III hypersensitivity), resulting in plasma leakage into body cavities, including the pericardial sac. Fluid accumulation leads to increased pressure in the pericardial sac and the development of cardiac tamponade, *i.e*. a potentially life-threatening complication. Although pericardial effusion is much less common than peritoneal or pleural effusion, its presence is clinically and diagnostically significant (14, 16, 17). In the case of animals that died as a result of disease and had previously exhibited cardiovascular symptoms, post-mortem and histopathological examinations revealed pathological changes within the cardiac muscle (12). The most commonly observed were foci of necrosis in the myocardium, petechiae on the epicardium and endocardium, the presence of fluid in the pericardial sac and numerous microthrombi within the cardiac muscle (12). Potential mechanisms leading to these changes include the accumulation of fibrin in the pericardial spaces and haemodynamic disturbances associated with blood stasis in the microcirculation, typical of an advanced FIP course.

The results of the present study show that fluid in the pericardial sac (pericardial effusion) was noted in 70.3% of cats among all the animals with confirmed effusive form of FIP, whereas cardiac tamponade was noted in 29.7% of animals. As mentioned in the introduction, cardiac symptoms accompanying FIP in cats have been increasingly observed in recent years. The authors’ observation contrasts with that of Tasker *et al*. (15), who reported that cardiac tamponade in the course of FIP is extremely rare and an exceptional complication observed only in isolated clinical cases. This discrepancy indicates that the disease evolves over time and that its clinical course may change. The presence of an increased amount of fluid in the pericardial sac can have serious haemodynamic consequences for patients with infectious peritonitis, especially if it leads to cardiac tamponade, as was the case in 10 cats under study. This indicates the need for a routine assessment of the pericardial cavity by ultrasound in cats with suspected FIP, especially those showing signs of cardiopulmonary failure. In addition, because NT-proBNP values in patients with tamponade were significantly higher, this concentration may be a biomarker for cardiac overload in the course of FIP.

Although fluid in the pericardial cavity found during ultrasound examination may suggest the effusive form of FIP, it should be emphasised that echocardiography alone is not sufficient to make a definitive diagnosis. To confirm FIP, a laboratory analysis of pericardial fluid is necessary. The present study used PCR testing of pericardial fluid to diagnose FIP. FIPogenic coronavirus strains were detected in fluid samples from all 37 cats. The final identification of the virus was based on the analysis of the melting temperatures of *S* gene fragment amplicons, which, for FIPogenic strains, ranged from 81.0 to 81.2°C. Similar melting point values were also obtained by other authors who detected the presence of FIPogenic virus strains in fluid from the abdominal cavity and the abdominal and chest cavities (1). The mutation determining the difference in melting temperature of amplicons obtained from intestinal and FIPogenic strains of the virus is the result of the substitution of adenine at position 23,531 of the *S* protein gene of intestinal strains with thymine or cytosine in FIPogenic strains, which consequently translates into the replacement of methionine with leucine at position 1,058 of the amino acid sequence of the protein. On this basis, these strains can be differentiated using the real-time PCR high-resolution melting SYBR Green technique (3).

This study has several limitations that should be acknowledged. Although the overall number of cats with effusive FIP was relatively high (n = 256), the subgroup presenting fluid accumulation exclusively in the pericardial sac comprised only 37 individuals. The relatively small size of this subgroup may have reduced the statistical power to detect subtle associations between breed, age or sex and the occurrence of pericardial effusion or cardiac tamponade. Therefore, the lack of statistically significant correlations should be interpreted with caution and does not exclude the presence of weak or moderate associations. In addition, the study was conducted at a single referral centre in Lublin, Poland. As a result, the study population reflects the regional feline demographic structure. The predominance of European mixed cats likely corresponds to their high prevalence in the local population rather than indicating a true breed predisposition. Consequently, the generalisability of the findings to other geographic regions or populations may be limited. Future multicentre studies involving larger and more geographically diverse feline populations are warranted to further clarify the epidemiology, pathophysiology and prognostic relevance of pericardial involvement in effusive FIP.

## Conclusion

Pericardial effusion may represent an underrecognised but clinically significant manifestation of effusive feline infectious peritonitis, frequently complicated by cardiac tamponade and myocardial overload. The findings of this study highlight the importance of routine echocardiographic assessment and molecular testing of pericardial fluid in cats with suspected FIP and cardiopulmonary signs. Further large-scale studies are warranted to better define the prevalence, pathophysiology and prognostic significance of cardiac involvement in the course of FIP and to assess the potential diagnostic value of biomarkers such as NT-proBNP.
